# Nonepiphyseal Giant Cell Tumor of the Rib: A Case Report

**DOI:** 10.1155/2012/745292

**Published:** 2012-10-16

**Authors:** Hippocrates Moschouris, Athanasios Marinis, Evanthia Bouma, Evangelos Karagiannis, Michalis Kiltenis, Marina Papadaki

**Affiliations:** ^1^Radiology Department, “Tzaneion” General Hospital, 1 Afentouli and Zanni STR, 18536 Piraeus, Greece; ^2^First Department of Surgery, “Tzaneion” General Hosptial, 1 Afentouli and Zanni STR, 18536 Piraeus, Greece

## Abstract

A case of a 32-year-old female patient with a giant cell tumor originating in the middle part of the left 10th rib is presented. On X-rays and CT, the tumor caused a well-defined osteolysis with nonsclerotic borders. On MRI, it exhibited intermediate signal intensity on T1 sequences and central high signal and peripheral intermediate signal on T2 sequences. On contrast-enhanced MR images both central and peripheral-periosteal enhancement was noted. Thanks to its small size (2 × 1.3 cm), the lesion was easily resected en bloc with a part of the affected rib. The patient is free of recurrence for 3 years after the operation.

## 1. Background

Giant cell tumor (GCT) is a relatively common, benign, locally aggressive tumor that typically presents in the 3rd and 4th decades of life, more commonly affecting women [1–3]. GCT usually affects the ends of the long bones and only rarely the ribs [1–3]. In this paper, we describe the spectrum of imaging findings in case of a GCT with a very unusual rib location. The histopathologic features of the lesion are also presented and our data are correlated with those of the literature.

## 2. Case Report

**Figure 1:**
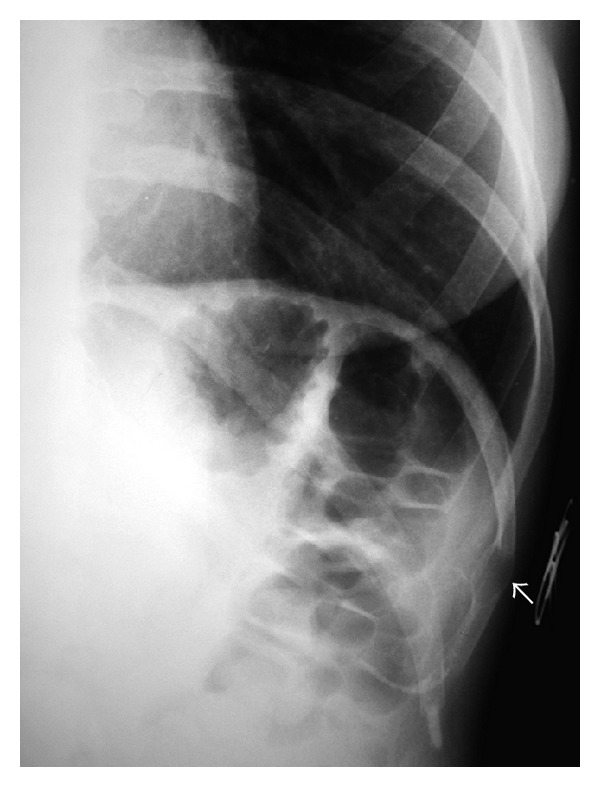
Conventional radiograph of the left lower ribs shows a well-circumscribed, ovoid lytic lesion (arrow) near the anterior aspect of the left tenth rib, causing thinning of the cortex and expansion of the bone.

**Figure 2:**
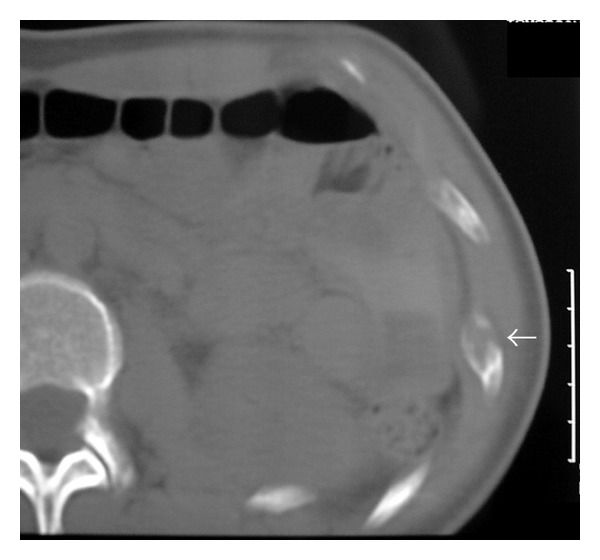
CT shows a soft tissue density lesion causing severe thinning of the bone cortex. The apparent area of cortical disruption (arrow) was not confirmed histologically and can be attributed to partial volume averaging.

**Figure 3:**
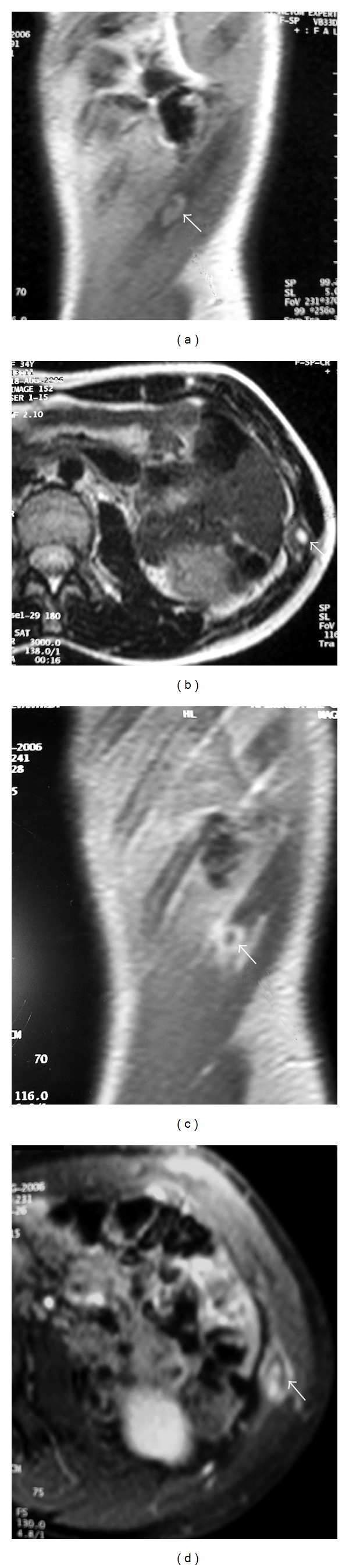
MR imaging of the tumor (arrows). (a) T1W, sagittal section shows an ovoid lesion with intermediate signal intensity, slightly higher than that of muscle. (b) T2W, axial section shows that the lesion has a high signal center and an intermediate signal periphery. After administration of paramagnetic contrast agent, there is both central and peripheral-periosteal enhancement. ((c) sagittal T1W + CM, (d) axial T1W FatSat + CM). The perilesional soft tissues show no signs of invasion. The findings are atypical.

**Figure 4:**
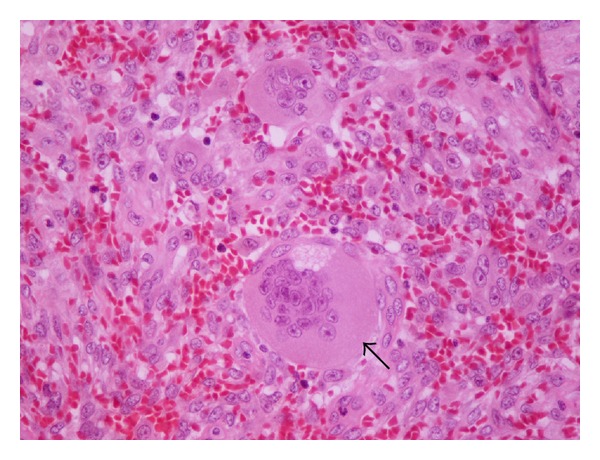
Histologic section of the tumor (Hematoxylin-Eosin ×400). Giant, multinucleated cells, lacking atypia or mitotic activity are present. The arrow points to one of these cells. Giant cells are disposed isolated or in small nests in abundant stroma. This is composed of spindle-shaped cells (some with storiform disposition, also lacking atypia or mitotic activity) and extravasated erythrocytes.

**Figure 5:**
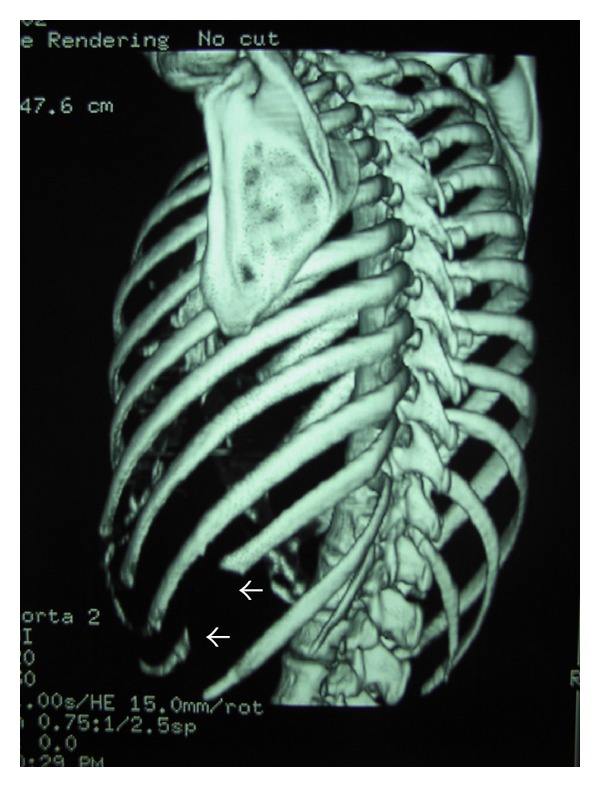
CT (3 years postoperatively), 3D reconstruction of the thoracic skeleton, showing the surgical defect at the middle part of the left 10th rib (arrow).

A 32-year-old female patient presented with recent onset of left lower chest pain. Her medical history was unremarkable. On the clinical examination, there was a localized tenderness at the middle part of the left tenth rib. She was initially referred for radiographs of the chest and left ribs. A well-circumscribed, ovoid lytic lesion was detected at the middle part of the left tenth rib, causing thinning of the cortex and expansion of the bone (Figure 1). Computed Tomography (CT) confirmed the presence of the lesion, which showed soft tissue density (Figure 2). On Magnetic Resonance Imaging (MRI), the lesion showed intermediate signal intensity on T1 sequences, while on T2 sequences a high signal center and an intermediate signal periphery were observed. After administration of paramagnetic contrast agent, both central and peripheral-periosteal enhancement was noted (Figure 3). Laboratory tests (including serum calcium, phosphorus, acid phosphatase, and alkaline phosphatase) were unremarkable. The tumor was resected en bloc with a small (4.5 cm long) part of the involved rib. No reconstruction of the chest wall with synthetic or autologous material was required. Pathologic examination confirmed the complete resection of the lesion, which measured 2 × 1.3 cm and a diagnosis of giant cell tumor of bone was obtained (Figure 4). Followup was performed with CT (Figure 5), MRI, and laboratory tests. She is free of recurrence for 3 years after the operation.

## 3. Discussion

Giant cell tumors are benign, though aggressive, neoplasms, accounting for 5 to 9 percent of all primary bony tumors [1]. Giant cell tumors are usually found in the long bones, most often the distal femur, proximal tibia, and distal radius [3]. GCT rarely arises in ribs (<1% in most series) [1, 2]. The posterior parts of the rib (head and tubercle) are most commonly affected, while involvement of the nonepiphyseal portions is very rare. Relatively, few case reports have been published with involvement of the anterior or middle part of the rib by GCT [4–8]. The studied tumors were generally large, with exophytic soft tissue component, causing a palpable lump or compressing and displacing adjacent organs (lung, liver, and breast).

On radiographs, GCT of the rib presents as a lytic lesion that may be eccentric and has well-defined, nonsclerotic margins. The lesion causes cortical thinning or even breakthrough and bone expansion [9]. CT clearly demonstrates the osseous lysis caused by the tumor. Matrix calcification is rare. Nevertheless, peripheral calcifications, internal septa (which can also be calcified), and cystic components have been described [4–7]. Disruption of the cortex and extension in the soft tissues can also be appreciated. MRI accurately delineates the tumor, demonstrates the involvement of the adjacent structures, and is helpful for the surgical planning. On the other hand, the MR features of GCT are not specific. Signal intensity is usually low or intermediate on T1W sequences and high on T2W sequences [9]. Inhomogeneous appearance is common, with signal voids (due to calcifications, hemosiderin, or vascular channels) and intensities indicative of hemorrhage at various stages [4, 7, 9]. Fluid-fluid levels are less common than in aneurysmal bone cyst [9]. The number of GCTs of the ribs imaged with MRI is limited. Most of them are larger lesions (compared to the herein presented tumor) with a more inhomogeneous appearance due to degeneration and haemorrhage. On bone scintigraphy, GCT is usually associated with markedly increased radionuclide activity and with a diffuse or “doughnut” pattern. These findings cannot facilitate either detection or characterization of GCT [10]. On FDG-PET, and contrary to the majority of benign bone tumors, GCT is associated with a high standardized uptake value (SUV), which does not differ significantly from the corresponding value of osteosarcoma [11]. Considerations regarding the radiologic differential diagnosis of the lesion of our paper are summarized in Table 1.

Serum acid phosphatase has been found to be increased in many cases of GCTs. It was therefore suggested that serum acid phosphatase could be used as a “tumor marker” for GCT and for monitoring treatment response and disease recurrence. It was also demonstrated that the level of this enzyme is correlated with the size of GCT [14]. In our case, serum acid phosphatase was normal preoperatively (probably due to the small size of the tumor) and remained at the same level postoperatively and during followup.

## 4. Conclusions

The remarkable features in our case of GCT are (a) the unusual location of the lesion at the middle part of the rib and (b) the small size of the lesion (in contrast to the majority of the other reported cases), permitting an easy and complete resection with no deformity of the chest wall postoperatively. Despite the multimodality imaging approach, the findings in this case of GCT were atypical and could not establish a preoperative diagnosis. Although remote, the possibility of a giant cell tumor may be included in the differential diagnosis when a relatively small, well-defined lytic lesion of a rib is encountered, even when this lesion is not located at the posterior part of the rib.

## Figures and Tables

**Table 1:** Differential diagnosis of a well-defined lytic lesion of the rib (based on [3, 9, 12, 13]).

Tumor name	Patient age	Rib location	Imaging pattern
Benign			

Fibrous dysplasia (monostotic)	Most patients: 20–30 years old	Common	May cause cortical thickening and exhibit amorphous calcification or ground glass appearance.
Enchondroma	2nd–5th decade of life	Common	Expansile lytic lesion, which may have calcified cartilaginous matrix. Usually located at the costochondral or costovertebral junction.
Eosinophilic granuloma	Most cases seen before the age of 30	Common	Solitary lytic lesion. A sclerotic margin may be present in the healing phase.
Brown tumor	Depends on the age of development of hyperparathyroidism	Rare	May be identical to GCT on imaging and histology. Laboratory tests should confirm hyperparathyroidism.
Aneurysmal bone cyst	Most cases seen before the age of 30	Rare	Expansile lesion which may exhibit bone destruction and extension into adjacent soft tissue. Fluid-fluid levels commoner than in GCT.
Simple bone cyst	Most cases seen before 20 yrs	Rare	Ovoid lytic lesion, often with a fine sclerotic margin.
Chondromyxoid fibroma	Less than 30 yrs	Rare	Well-marginated masses with scalloped sclerotic borders. No internal calcification. Possible cortical expansion.

Malignant			

Metastasis	Commoner after 50 yrs	Common	Well- or ill-defined osteolysis without sclerotic rim. Most common primaries: lung and breast.
Solitary myeloma	Mean age: 50 yrs	Common	Well-defined, “punched out” lytic lesion, which may cause expansion. No sclerotic margin.
Chondrosarcoma (low grade)	Most cases seen after 50 yrs	Common	Better differentiated tumors are well defined. Arc-like, stippled, or amorphous calcification is common.
